# Structure of metallochaperone in complex with the cobalamin-binding domain of its target mutase provides insight into cofactor delivery

**DOI:** 10.1073/pnas.2214085120

**Published:** 2023-02-14

**Authors:** Francesca A. Vaccaro, David A. Born, Catherine L. Drennan

**Affiliations:** ^a^Department of Chemistry, Massachusetts Institute of Technology, Cambridge, MA 01239; ^b^Graduate Program in Biophysics, Harvard University, Cambridge, MA 01238; ^c^Department of Biology, Massachusetts Institute of Technology, Cambridge, MA 01239; ^d^HHMI, Massachusetts Institute of Technology, Cambridge, MA 01239

**Keywords:** cofactor delivery, metalloenzyme maturation, G-protein chaperone, cobalamin, signal transduction

## Abstract

G-protein metallochaperone MeaB in bacteria [methylmalonic aciduria type A (MMAA) in humans] is responsible for facilitating the delivery of adenosylcobalamin (AdoCbl) to methylmalonyl-CoA mutase (MCM), the only AdoCbl-dependent enzyme in humans. Genetic defects in the switch III region of MMAA lead to the genetic disorder methylmalonic aciduria in which the body is unable to process certain lipids. Here, we present a crystal structure of *Methylobacterium extorquens* MeaB bound to a nonhydrolyzable guanosine triphosphate (GTP) analog guanosine-5′-[(β,γ)-methyleno]triphosphate (GMPPCP) with the Cbl-binding domain of its target mutase enzyme (*Me*MCM_cbl_). This structure provides an explanation for the stimulation of the GTP hydrolyase activity of MeaB afforded by target protein binding. We find that upon MCM_cbl_ association, one protomer of the MeaB dimer rotates ~180°, such that the inactive state of MeaB is converted to an active state in which the nucleotide substrate is now surrounded by catalytic residues. Importantly, it is the switch III region that undergoes the largest change, rearranging to make direct contacts with the terminal phosphate of GMPPCP. These structural data additionally provide insights into the molecular basis by which this metallochaperone contributes to AdoCbl delivery without directly binding the cofactor. Our data suggest a model in which GTP-bound MeaB stabilizes a conformation of MCM that is open for AdoCbl insertion, and GTP hydrolysis, as signaled by switch III residues, allows MCM to close and trap its cofactor. Substitutions of switch III residues destabilize the active state of MeaB through loss of protein:nucleotide and protein:protein interactions at the dimer interface, thus uncoupling GTP hydrolysis from AdoCbl delivery.

## Significance

Guanosine triphosphate hydrolyase (GTPase) metallochaperones enable the delivery of metal cofactors to client proteins in host organisms that range from the pathogen *Helicobacter pylori* to humans. The combination of metal with protein facilitates critical cellular reactions. For example, nickel-dependent urease provides the requisite buffering capacity that allows for *H. pylori* to survive in the acidic human stomach. Human adenosylcobalamin-dependent methylmalonyl-CoA mutase is a vital metabolic enzyme whose impairment leads to disease. Overexpression and maturation of mutases and metal-dependent hydrogenases are of interest for industrial applications such as the production of carbon fixation cycles and biofuel cells. In short, understanding the molecular basis of metallochaperone function has numerous applications that extend from the development of *H. pylori* therapies to human health to metalloenzyme overexpression.

Metalloproteins are ubiquitous in biology, accounting for about 30 to 50% of all proteins. The catalysis of chemically challenging reactions in biology often requires a metallocofactor. This catalytic prowess can come at a price, however, as metal ions can be toxic to the cell. To prevent toxicity and to afford proper metalloprotein maturation, specialized proteins known as metallochaperones are employed for metallocofactor transport and/or delivery (1–3). Guanine nucleotide-binding proteins (G-proteins) belonging to the signal recognition particle, MinD, and BioD (SIMIBI) class of P-loop NTPases are one important class of metallochaperones (4). These proteins include UreG (5, 6), which is involved in the assembly of the nickel metallocofactor of urease; HypB (7, 8), which is involved in incorporating Ni^2+^ ions into hydrogenase; and MeaB [methylmalonic aciduria type A (MMAA) in humans], which is involved in the delivery of coenzyme B_12_ (5′-deoxyadenosylcobalamin or AdoCbl) to methylmalonyl-CoA mutase (MCM) and related mutases (9–14). Without these metallochaperones, metalloprotein function is impaired (15). In humans, mutations to MCM, MMAA, or any of the other B_12_ trafficking proteins result in inborn errors of metabolism (16).

**Fig. 1.**
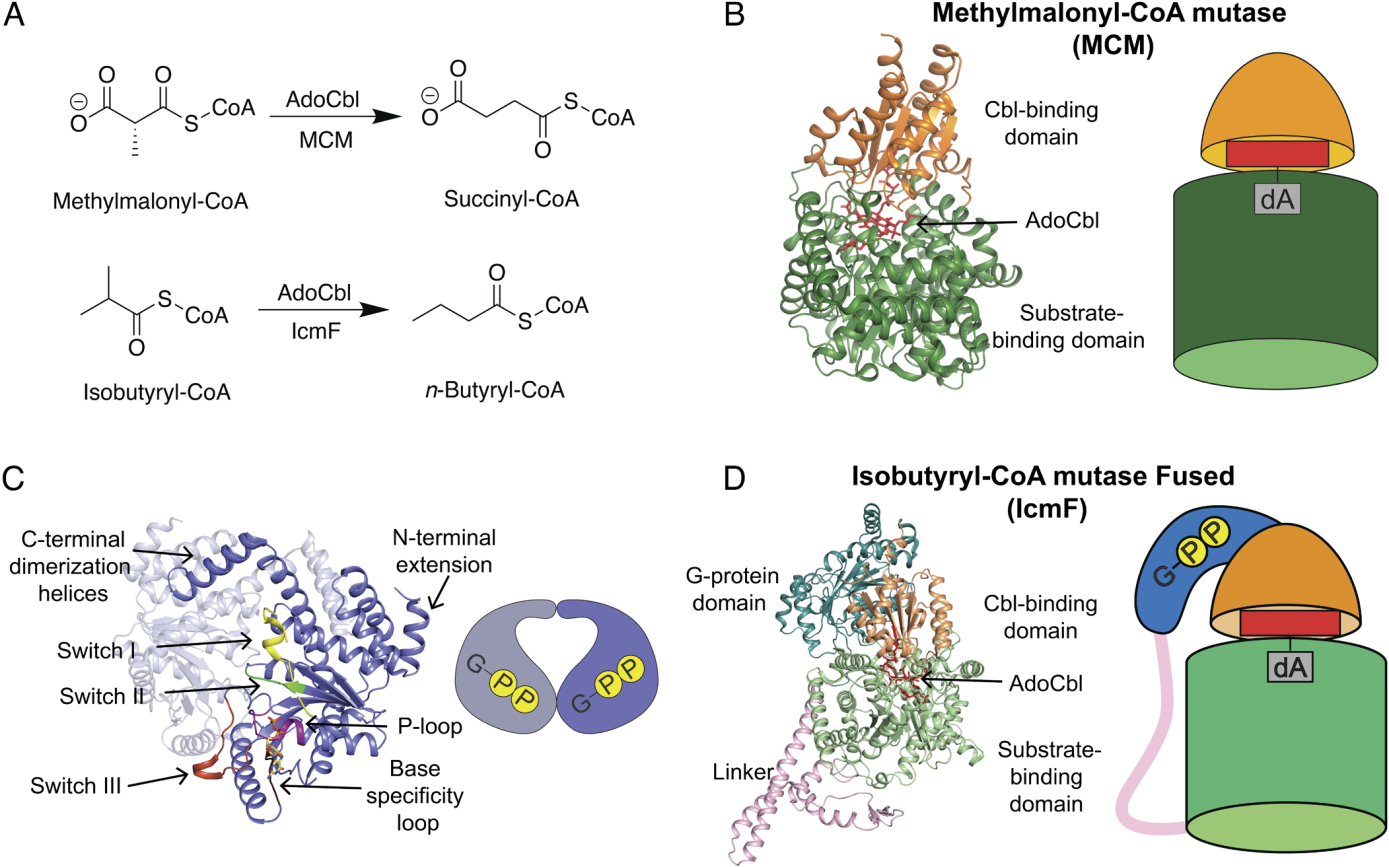
AdoCbl-dependent mutases require G-protein chaperones for maturation. (*A*) Reaction catalyzed by MCM (*Top*) and a reaction catalyzed by IcmF (*Bottom*). (*B*) The overall structure of the monomer of MCM from *P. shermanii* bound to AdoCbl (PDB 4REQ). The AdoCbl (red sticks) is bound at the interface of the Cbl-binding domain (light orange) and substrate-binding domain (green). The cartoon representation of MCM has the same coloring as the ribbon structure. (*C*) Signature motifs of the G3E P-loop GTPases shown on MeaB (slate) from *M. extorquens* bound to GDP (PDB 2QM7). The P-loop (purple, residues (res. 62 to 70) interacts with the phosphates of the nucleotide. The base specificity loop (brown, res. 200 to 207) interacts with the guanosine base. The switch I (yellow, res. 92 to 108), switch II (green, res. 154 to 158) and switch III (red orange, res. 177 to 188) function as switches signaling the GTP hydrolysis event. (*D*) The overall structure of one monomer of the dimeric IcmF from *Cuprividius metallidurans* bound to GDP and AdoCbl (PDB 4XC6) contains the Cbl-binding domain (light orange) and substrate-binding domain (light green) of the mutase on same polypeptide chain as the G-protein domain (teal) connected by a polypeptide linker (pink). AdoCbl (red sticks) is bound in the active site at the interface of the Cbl-binding and substrate-binding domains. The cartoon representation of IcmF has the same coloring as the ribbon structure.

This study focuses on MeaB from the bacteria *Methylobacterium extorquens* (17)*,* which facilitates the delivery of AdoCbl to apo MCM and also assists in the removal of damaged cob(II)alamin from holo MCM. MCM catalyzes the 1,2-rearrangement of (*R*)-methylmalonyl-CoA to succinyl-CoA (Fig. 1*A*), a necessary step in the metabolism of odd-chain fatty acids, cholesterol, and branched amino acids (18). The AdoCbl cofactor is essential for this chemically challenging carbon skeletal rearrangement. Homolytic cleavage of the carbon–cobalt bond of AdoCbl generates cob(I)alamin and a highly reactive 5′-deoxyadenosyl radical species, which abstracts a hydrogen atom from substrate to initiate this radical-based reaction. Upon reaction completion, the product radical abstracts a hydrogen atom back from 5′-deoxyadenosine and the AdoCbl is reformed. If however the cob(II)alamin is oxidized to cob(III)alamin, AdoCbl cannot be reformed, and the cob(III)alamin must be removed from the enzyme and replaced with a new AdoCbl. MeaB facilitates both the removal of the damaged cofactor and the insertion of a new cofactor. Importantly, MeaB does so without directly binding the cobalamin (Cbl) cofactor. In a process that requires guanosine triphosphate (GTP) hydrolysis, MeaB enables the AdoCbl, which was generated by an adenosyltransferase (ATR) from cob(II)alamin and ATP, to be transferred from ATR to MCM.

To understand the molecular basis by which MeaB performs this chaperoning function, our lab and others have determined structures of bacterial MeaB and its human counterpart MMAA (12, 19–21) as well as structures of bacterial and human MCMs (22, 23). Human MCM is a homodimer, whereas bacterial MCMs are commonly heterodimers with one inactive subunit and one active subunit (12, 23). Active subunits, as depicted in the structure of MCM from *Propionibacterium shermanii,* have a characteristic Rossmann domain that binds AdoCbl and a triose-phosphate isomerase (TIM) barrel domain to bind the substrate (Fig. 1*B*) (23–25). Enzyme activity requires a “closed” state in which the Ado moiety of AdoCbl is positioned within the substrate-binding TIM barrel, whereas cofactor delivery would require an “open” state in which the Cbl-binding Rossmann domain is displaced from the TIM barrel. Presumably, MeaB assists in either or both the opening and closing of MCM.

The structure determination of MeaB revealed a common G-domain fold composed of regularly recurring α-β units with the β-strands forming a central β-sheet surrounded on both sides by α-helices (Fig. 1*C*) (19). Consistent with its classification as a P-loop NTPase, MeaB contains a P-loop (residues 62 to 70), a base-specificity loop (residues 200 to 207), and two conserved switch regions for signal transduction: switch I (residues 92 to 108) and switch II (residues 154 to 158) (Fig. 1*C*) (19, 20, 26–28). MeaB and MMAA also contain an N-terminal extension domain (12, 19), a C-terminal extension domain involved in dimerization, and a switch III region (residues 177 to 188) (Fig. 1*C*) (21). The switch III region was identified by the Banerjee lab through the investigation of residues whose mutation is associated with methylmalonic aciduria, an inborn error of metabolism (29). In vitro, they found that the substitution of switch III residues Lys188, Gln185, or Asp182 with alanine led to an uncoupling of GTP hydrolysis from AdoCbl transfer (21). Thus, prior work has informed our understanding of which regions of MeaB (switches I, II, and III) are important for function. All structures, regardless of whether MeaB was bound to guanosine diphosphate (GDP), the nonhydrolyzable analog of GTP, guanosine-5'-[(β,γ)-imido]triphosphate (GMPPNP), or no nucleotide, showed that the switch I and II regions were buried at the MeaB dimer interface, and thus inaccessible, and that the switch III residues were more than 10 Å away from the nucleotide phosphates, too far to explain how they might be involved in the uncoupling of GTP hydrolysis from cofactor insertion (Fig. 1*C*) (19, 21). The structures of MeaB alone thus indicated that additional conformational states of the G-protein must exist.

Most G-protein metallochaperones are poor guanosine triphosphate hydrolyases (GTPases) [for example, MeaB alone has a k*cat* of 0.039 ± 0.003 min^−1^ (30)] in the absence of their target protein. In fact, many GTPases have GTPase-accelerating proteins (GAP) that stimulate GTP hydrolysis through binding. In the case of MeaB, GTP hydrolysis is increased ~100-fold in the presence of MCM, again suggesting that MeaB may undergo a conformational change upon binding MCM (9, 12). No structure has been obtained of MeaB bound to MCM, but a structure of a natural fusion protein of a MeaB-like G-protein domain with a mutase enzyme has been solved (Fig. 1*D*). This protein, isobutyryl-CoA mutase fused (IcmF), interconverts isobutyryl-CoA and *n*-butyryl-CoA (Fig. 1*A*), as well as pivalyl-CoA and isovaleryl-CoA (31). The IcmF structure revealed a monomeric G-protein domain wrapped around the Cbl-binding domain of the mutase with the switch I and II regions contacting the nucleotide-binding site and the switch I region making direct contacts to the Cbl-binding domain (32). However, it was unclear how this fused protein structure could be interpreted in terms of a dimeric standalone MeaB (32). Additionally, the switch III region was solvent exposed in this structure and did not make any protein:protein contacts or protein:nucleotide contacts (32), indicating that a structure of the catalytically relevant state of MeaB was still missing.

Since the large 123-kDa fusion protein did not provide all of the requisite structural data, we turned to a minimal system. We engineered a minimal mutase system consisting of solely the Cbl-binding domain of *M. extorquens* MCM (*Me*MCM_cbl_), removing the substrate-binding TIM barrel domain. Using this minimal mutase system and a nonhydrolyzable GTP analog guanosine-5′-[(β,γ)-methyleno]triphosphate (GMPPCP), we have trapped a conformation of a G-protein chaperone that has not been previously observed. This conformation reveals an ordered nucleotide-binding site with switch III residues interacting across the dimer interface, providing a molecular route for signaling.

## Results

### The Cbl-Binding Domain of *Me*MCM Provides a Minimal Model for the Investigation of MeaB’s GTPase Activity in the Presence of MeaB’s Target.

**Fig. 2.**
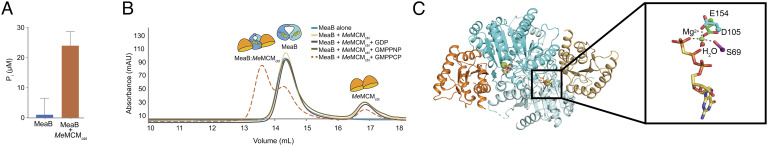
*Me*MCM_cbl_ increases GTP hydrolysis of MeaB and can form a stable complex with MeaB allowing for structure determination. (*A*) MeaB alone (blue) or MeaB with *Me*MCM_cbl_ (orange) was incubated with 500 µM of GTP for 30 min at 22 °C. MeaB alone produced 0.95 µM of P_i_ and MeaB in the presence of *Me*MCM_cbl_ produced 24.0 µM of P_i_. The amount of inorganic phosphate produced by MeaB in the presence of *Me*MCM_cbl_ is 25-fold greater. The no enzyme control was subtracted from both conditions. The reactions were performed in triplicate; error bars represent the 95% CI. (*B*) The S200 10/300 size exclusion chromatograms indicate that the formation of a stable complex only occurs in the presence of GMPPCP (dashed orange). The traces of MeaB alone (blue), MeaB and *Me*MCM_cbl_ with no G-nucleotide (yellow), MeaB and *Me*MCM_cbl_ with GDP (black), and MeaB and *Me*MCM_cbl_ with GMPPNP (green) indicate no stable complex formation. (*C*) The asymmetric unit of the MeaB:*Me*MCM_cbl_:GMPPCP structure contains the MeaB homodimer (chain A and chain B, cyan and pale cyan, respectively) bound to two molecules of GMPPCP (yellow sticks) and two Mg^2+^ ions (green spheres) and two apo *Me*MCM_cbl_ protomers (orange and tan). Insert: Mg^2+^ is coordinated by a water (red sphere), the oxygen atoms of Ser69, Asp105, Glu154, and the β- and γ-phosphate groups of GMPPCP.

To assess the putative GTPase activating properties of the *Me*MCM_cbl_, we incubated MeaB with apo *Me*MCM_cbl_ and 500 µM GTP. We monitored the amount of inorganic phosphate (P_i_) produced. In the same amount of time, MeaB in the presence of *Me*MCM_cbl_ produced 25 times more P_i_ than MeaB alone (Fig. 2*A*). This observation validates the ability of the Cbl-binding domain alone to activate the GTPase activity of MeaB. To test whether apo *Me*MCM_cbl_ and MeaB form a stable complex, analytical size exclusion chromatography was used. Both MeaB and *Me*MCM_cbl_ migrated independently with apparent molecular weights of 75 kDa and 40 kDa, respectively, consistent with both proteins forming homodimers. A mixture of MeaB and apo *Me*MCM_cbl_ preincubated with GMPPCP eluted with a higher molecular weight species in addition to the individual protein dimer peaks (Fig. 2*B*). The higher molecular weight species had an apparent molecular weight of 110 kDa, consistent with one MeaB dimer and one *Me*MCM_cbl_ dimer. Sodium dodecyl sulfate polyacrylamide gel electrophoresis (SDS-PAGE) analysis confirmed the coelution of MeaB and *Me*MCM_cbl_ from this peak (*SI Appendix*, Fig. S1), indicating complex formation. The presence of the individual dimer peaks eluting after the high molecular weight species suggests an equilibrium between the complex and its protein components. Interestingly, the complex requires prebound GMPPCP for its formation. No complex was observed with GDP or GMPPNP or in the absence of nucleotide.

### MeaB Uses an Alternative Conformation to Bind the Apo Form of MCM’s Cbl-Binding Domain.

To investigate how MeaB interacts with an apo form of the Cbl-binding domain of *Me*MCM in the absence of the substrate-binding TIM barrel, we solved the structure of MeaB in the presence of apo *Me*MCM_cbl_ and GMPPCP to 2.72 Å resolution using molecular replacement (*SI Appendix*, Table S1). The final model contained a MeaB dimer (chains A and B) and two *Me*MCM_cbl_ protomers (chains C and D) in the asymmetric unit (Fig. 2*C*). As reported previously, MeaB has a characteristic G-domain fold that is typical of G-proteins (19), and the mutase Cbl-binding domain has the characteristic Rossmann fold (23). Only the first 18 residues of each of the *Me*MCM_cbl_ protomers are disordered, which is not surprising given that these residues represent a linker region between the domains when *Me*MCM_cbl_ is part of a full-length enzyme. There is one Mg^2+^ ion and one GMPPCP molecule associated with each of the MeaB protomers (Fig. 2*C* and *SI Appendix*, Fig. S2). The Mg^2+^ ion is coordinated by six oxygens: an oxygen atom from the β- and γ- phosphate groups of GMPPCP, one water, and the side chains of Ser69, Asp105, and Glu154 (Fig. 2*C*).

**Fig. 3.**
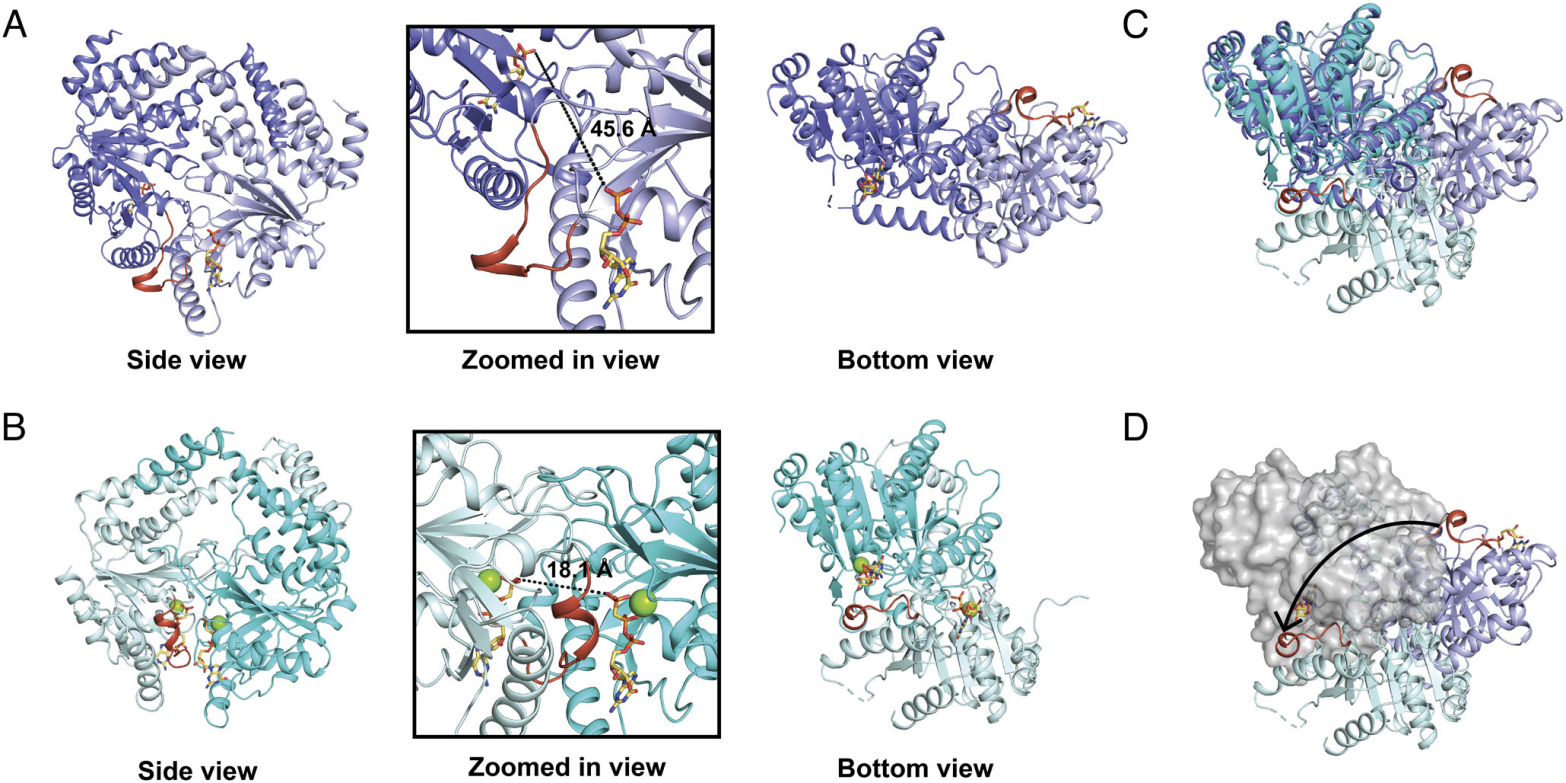
The structure of the MeaB:*Me*MCM_cbl_:GMPPCP complex reveals an active MeaB conformation. (*A*) MeaB bound to GDP (PDB 2QM7) from side view (same view shown in Fig. 1) and bottom view. Zoomed in panel shows distances between bound nucleotides in this previously observed MeaB conformation. The switch III region is in red orange. (*B*) The MeaB dimer rearrangement from MeaB:*Me*MCM_cbl_:GMPPCP shown in side view and bottom view. Relationships between side and bottom views are shown in *SI Appendix*, Fig. S8. The upper protomers of both bottom views (here and in *A*) are oriented identically. Zoomed in panel shows distances between bound nucleotides in this active MeaB conformation. (*C*) Bottom views superimposed. Colored as in *A* and *B*. (*D*) Bottom view in which both upper protomers are aligned and are shown as a gray surface, the lower protomer of MeaB:*Me*MCM_cbl_:GMPPCP is in pale cyan, and lower protomer of MeaB bound to GDP is in light purple. The switch III region (red orange) moves from being solvent exposed when MeaB is bound to GDP to being at the interdimer interface of MeaB in the MeaB:*Me*MCM_cbl_:GMPPCP complex. The arrow indicates the conformational change required to form the MeaB:*Me*MCM_cbl_ complex.

The comparison of the overall conformation of the MeaB dimer as it exists in the MeaB:*Me*MCM_cbl_:GMPPCP complex to the previous structures of apo MeaB or GDP-bound MeaB (19, 21) reveals an alternative arrangement of MeaB protomers (Fig. 3 *A* and *B*). When aligning one of the MeaB protomers of each structure, there is almost a 180° rotation of the other protomer needed to alter the conformation of MeaB that is observed on its own to the conformation observed in the MeaB:*Me*MCM_cbl_ complex structure (Fig. 3 *C* and *D*). This movement relocates the switch III region such that it forms new interactions with the other protomer of MeaB and creates a new interface that buries the MeaB nucleotide-binding site.

### Active Conformation of MeaB Creates a Fully Ordered Nucleotide Binding Site Using Both Protomers.

**Fig. 4.**
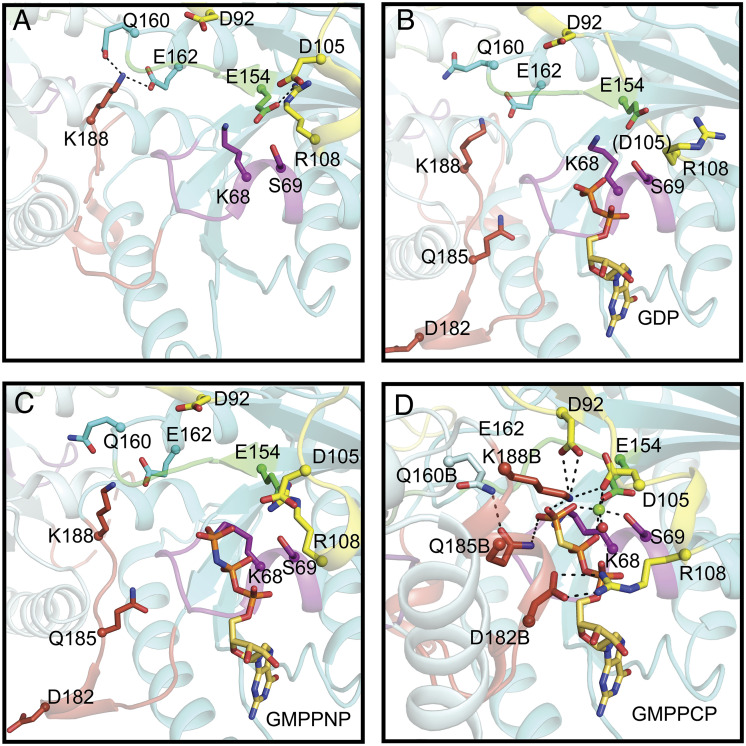
Comparison of the interactions of nucleotide-binding sites in MeaB structures with MeaB:*Me*MCM_cbl_:GMPPCP structure. (*A*) Nucleotide-free MeaB (PDB 2QM8). P-loop (purple), switch I (yellow), switch II (green), switch III (red orange), additional residues that are outside of the motifs that undergo conformational rearrangements (cyan). Base specificity loop interactions are shown in *SI Appendix*, Fig. S4 instead of here for clarity. (*B*) GDP-bound MeaB (PDB 2QM7). (*C*) GMPPNP-bound MeaB (PDB 4JYB). (*D*) GMPPCP-bound MeaB in the presence of *Me*MCM_cbl_ (this work). The residues shown in sticks undergo nucleotide-dependent interactions that contribute to the conformational changes necessary to increase GTP hydrolysis and communicate with the mutase. Lys188B and Gln185B are ~2.1 Å and ~3.5 Å from the γ-phosphate of GMPPCP, respectively. Lys188B is also ~3.4 Å away from Asp92. The “B” label indicates residues from MeaB chain B.

A comparison of this new MeaB structure with previous MeaB structures and the IcmF structure shows both previously observed and unreported interactions between the nucleotide and chaperone domains (19, 21, 32). In particular, the base-specificity loop and guanine base are the same between structures (*SI Appendix*, Figs. S3 and S4). The interactions of the backbone atoms of the P-loop and the nucleotide phosphate groups are also similar (*SI Appendix*, Figs. S3 and S4). Previous structures of MeaB do not have a Mg^2+^ ion bound (Fig. 4 *A*–*C*) but from comparison with the Mg^2+^-GDP-bound IcmF structure (32) (*SI Appendix*, Fig. S5) and the structure of HypB (8), three residues, Ser69, Asp105, and Glu154 were predicted to coordinate the Mg^2+^ ion in MeaB (19). Our MeaB:*Me*MCM_cbl_:GMPPCP structure confirms this prediction; as mentioned above, side chains of Ser69, Asp105, and Glu154 provide three coordinating oxygens, which together with oxygen atoms from the β- and γ-phosphate groups of GMPPCP and one water complete the Mg^2+^ ion’s coordination sphere (Figs. 2*C* and 4*D*). A comparison with previous MeaB structures shows that both Asp105 and Glu154 become ordered and/or repositioned to coordinate the bound Mg^2+^ ion (Fig. 4). This movement of Glu154 to coordinate the Mg^2+^ ion and to interact with Lys188B leads to a series of conformational rearrangements. First, Glu154 movement breaks the interaction between Glu154 and Arg108, freeing the Arg108 side chain (Fig. 4). The side chain of Arg108 undergoes a substantial rearrangement such that it can coordinate the α-phosphate group of GMPPCP (Fig. 4*D*). In this conformation, Arg108 also forms a salt bridge with Asp182 from the second protomer (Asp182B of switch III), playing a role similar to that of an Arg finger, a common functional motif in the protein superfamily that includes GTPases (33, 34).

An important consequence of the ~180° MeaB protomer rearrangement described above is that the switch III residues of chain B are now positioned to contact the nucleotide-binding site of chain A. In addition to Asp182, switch III residue Gln185 of chain B now contacts the β-phosphate of GMPPCP in chain A (Fig. 4*D*). Previously, Gln185 was either disordered (Fig. 4*A*) or made no observable interactions (Fig. 4 *B* and *C*). Finally, switch III residue Lys188 from chain B rearranges to coordinate the γ-phosphate group of the nucleotide and interacts with switch I residue Asp92 and switch II residue Glu154 (Fig. 4*D*). In previous MeaB structures (19, 21), Lys188 formed hydrogen bonds with Gln160 and Glu162 but showed no nucleotide interactions and no interactions with other switch residues (Fig. 4 *A*–*C*). Thus, this structure provides a molecular explanation for the involvement of switch III in nucleotide-dependent signaling. It also provides the previously undetermined snapshot of MeaB prior to GTP hydrolysis, revealing formerly unknown interactions between the protein and the Mg^2+^ ion and the protein and the nucleotide.

Due to the increased number of interactions made by the protein to the GMPPCP in this alternative MeaB conformation (Fig. 4), it would appear that this MeaB conformer represents the “active state” of this GTPase and the structure of the conformer previously solved represents the “inactive state.” The “active conformer” is stabilized by interactions across the dimer interface (Asp92A–Lys188B; Arg108A–Asp182B; Glu154A–Lys188B; Gln160A–Gln185B) and by interactions of switch III residues with the GTP (Asp182B–α-phosphate; Gln185B–γ-phosphate; Lys188B–γ-phosphate) (*SI Appendix*, Table S2).

### Switch I Region Makes Up the MeaB:*Me*MCM _cbl_ Interface.

**Fig. 5.**
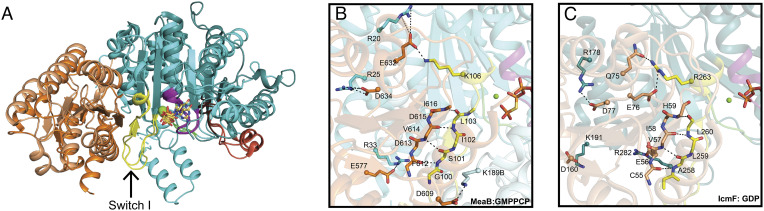
Interactions at the interface of the MeaB and *Me*MCM_cbl_ in the MeaB:*Me*MCM_cbl_:GMPPCP complex. (*A*) Superimposition of *Me*MCM_cbl_ (orange) and one protomer of MeaB (cyan) from the MeaB:*Me*MCM_cbl_:GMPPCP complex on the Cbl-binding domain (light orange) and G-protein domain (teal) from IcmF (PDB 4XC6) shows a similar interface between domains. Additionally, the conserved regions of the G-protein are in similar orientations. Coloring: Base loop (dark brown), P-loop (purple), switch I (yellow and labeled with an arrow), switch II (green), and switch III (red orange). (*B*) The interface between MeaB (cyan with switch I region in yellow) and *Me*MCM_cbl_ (orange) primarily consists of the hydrogen bonding of two β-strands, one from MeaB (switch I residues 100 to 103 in yellow) and one from *Me*MCM_cbl_ (residues 612 to 616 in orange). The side chains of the β-strands are omitted for simplicity. Additionally, the interface has five observed salt bridges, K189B:D609, K106:D632, R33:E577, R25:D634, and R20:D632. The “B” indicates residues from MeaB chain B. (*C*) The interface between the G-protein domain (teal with switch I in yellow) and the Cbl-binding domain (light yellow) of IcmF (PDB 4XC6) primarily consists of hydrogen bonding to two β-strands, one from the G-protein domain (switch I residues 258 to 260 in yellow) and one from the Cbl-binding domain (residues 55 to 59 also yellow), shown as sticks. The side chains of the β-strands are omitted for simplicity. Additionally, the interface has five observed salt bridges, R178:D77, K191:D160, R263:Q75, R263:E76, and R282:E56.

Using our structure of Mg^2+^-GMPPCP-bound MeaB:*Me*MCM_cbl_, we were able to investigate how the binding of *Me*MCM_cbl_ can shift the conformational equilibrium of MeaB from an “inactive conformer” to an “active conformer.” We find that one protomer of MeaB contacts the edge of the Rossmann domain of *Me*MCM_cbl_ in an analogous fashion as was observed in the structure of IcmF (Fig. 5*A*) (32). Residues (99 to 107) of the switch I region that were disordered in the initial MeaB structure (19) become ordered and form the majority of the interface with *Me*MCM_cbl_, explaining the molecular basis of switch I signaling. Notably, MeaB switch I residues 100 to 103 form a β-strand that runs parallel to, and hydrogen bonds with, a β-strand of the Rossmann domain of *Me*MCM_cbl_ (residues 612 to 616). This interaction extends the Rossmann domain’s β-sheet from five strands to six (Fig. 5 and *SI Appendix*, Table S3). With *Me*MCM_cbl_ now forming a continuous β-sheet with MeaB, the chaperone and target protein would be expected to move as a rigid body, which was observed for IcmF previously (32). Further anchoring MeaB and *Me*MCM_cbl_ together are five salt bridges, Lys189B:Asp609, Lys106:Asp632, Arg33:Glu577, Arg25:Asp634, and Arg20:Asp632 (Fig. 5*B* and *SI Appendix*, Table S4); all but one of them are formed by chain A of MeaB. The one notable exception is the salt bridge consisting of Lys189B (a switch III residue) of MeaB and Asp609 of *Me*MCM_cbl_.

Although it was unclear at the time how relevant the structure of IcmF would be to a dimeric standalone MeaB system (32), we now see that much of the information provided by the IcmF structure was relevant. We find that it is one protomer of MeaB (chain A) that makes most of the interactions to *Me*MCM_cbl_ (1150 Å^2^ buried by chain A and 225 Å^2^ buried by chain B) and that the interactions made, including those made by switch I residues, are the same in MeaB:MCM as in IcmF (Fig. 5), where IcmF, and its monomeric chaperone domain, was unsuccessful as a model system in providing insights into the importance of the switch III residues, and we now see why. It is the second protomer of MeaB (chain B) that contributes switch III residues to the chaperone:target interface and to the nucleotide-binding site.

### Superimposition of the Minimal System on Full-Length Mutase Structures Reveals MeaB:Mutase Clashes When the Cbl-Binding Domain Is Positioned for Catalysis.

**Fig. 6.**
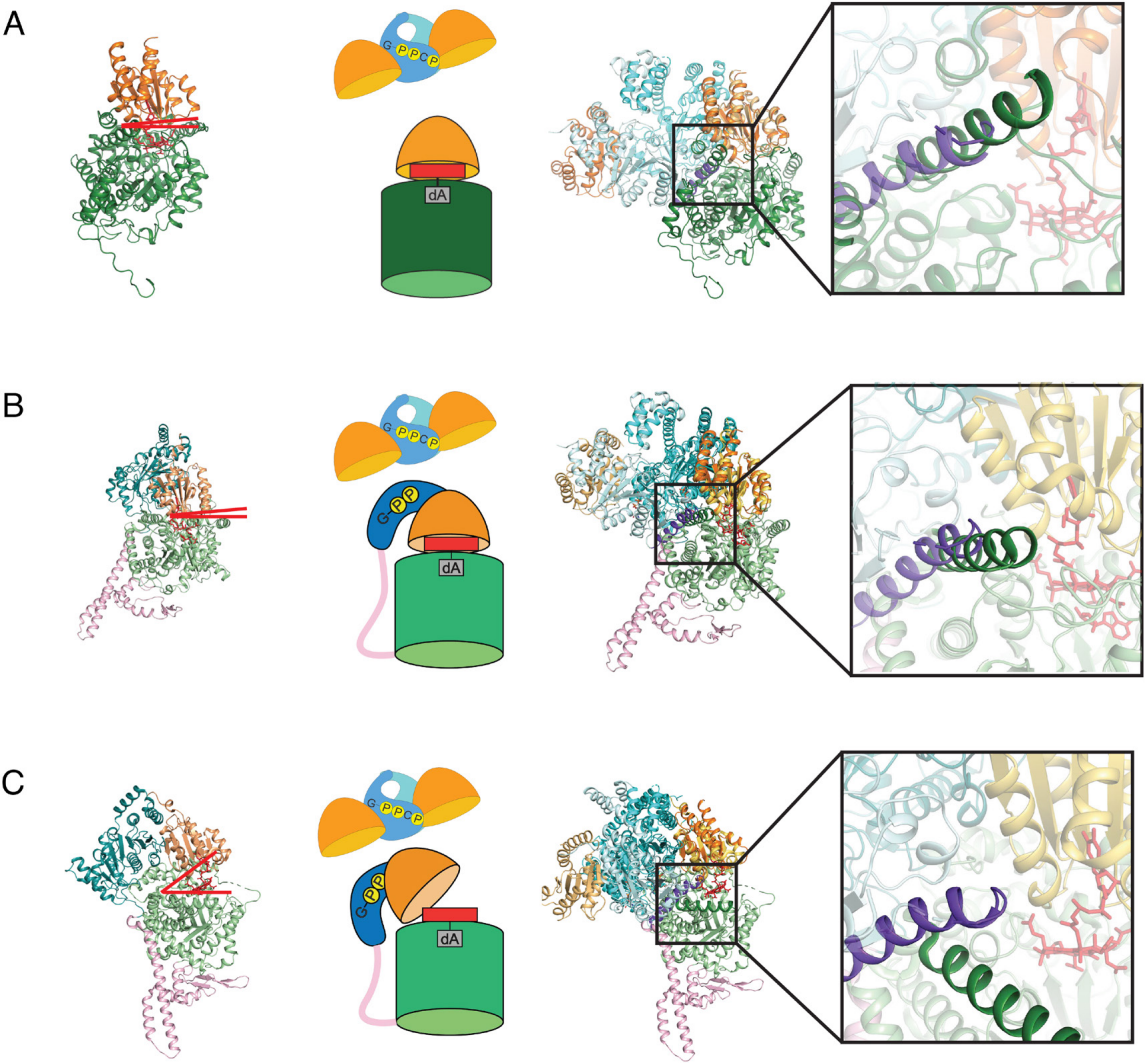
Superimposition of MeaB:*Me*MCM_cbl_:GMPPCP onto full-length mutase structures. (*A*) (*Left*) Ribbon drawing of *Ps*MCM (PDB 2REQ) with substrate-binding domain (dark green) and Cbl-binding domain (light orange). Red lines indicate that there is no gap between domains; the Cbl is positioned into the barrel for catalysis. (*Middle*) Cartoon of MeaB:*Me*MCM_cbl_ and an active protomer of *Ps*MCM. (*Right*) Overlay of MeaB:*Me*MCM_cbl_ with the *Ps*MCM structure with *Inset* showing clash between an alpha helix of MeaB (residues 206 to 228 in purple) and the substrate binding domain (residues 441 to 459 in dark green) of *Ps*MCM. (*B*) (*Left*) IcmF (PDB 4XC6) with substrate-binding domain (light green), Cbl-binding domain (light orange), G-protein domain (teal), and linker (pink). Red lines indicate that there is no gap between domains; the Cbl is positioned into the barrel for catalysis. (*Middle*) Cartoon of MeaB:*Me*MCM_cbl_ and an active IcmF protomer. (*Right*) Overlay of MeaB:*Me*MCM_cbl_ with the closed IcmF structure with *Inset* showing a clash between an alpha helix of MeaB (residues 206 to 228 in purple) and the substrate binding domain (residues 975 to 995 in dark green) of IcmF. (*C*) (*Left*) Ribbon drawing of open IcmF (PDB 4XC6) with colors indicated in (*B*). Red lines indicate a gap between domains is available for Cbl insertion. (*Middle*) Cartoon of MeaB:*Me*MCM_cbl_ and an inactive protomer of IcmF. (*Right*) Overlay of MeaB:*Me*MCM_cbl_ with the open protomer of IcmF with *Inset* showing that the helix of the substrate binding domain of IcmF (dark green) does not clash with the helix of MeaB (purple) when the mutase is “open”.

To investigate how the “active conformation” of MeaB, as observed in our minimal system, would interact with a full-length mutase, we superimposed *Me*MCM_cbl_ onto the Cbl-binding domains of previously solved structures of full-length mutases *P. shermanii* MCM (*Ps*MCM) (23) and IcmF (32). These Cbl-binding domains have high structural similarity: RMSD of 1.195 Å (Cα only) for *Me*MCM_cbl_ with the *Ps*MCM and 1.193 Å and 1.180 Å (Cα only) for *Me*MCM_cbl_ with dimeric IcmF (*SI Appendix*, Fig. S6). When MeaB:*Me*MCM_cbl_ is superimposed on the full-length *Ps*MCM using the Cbl-binding domains for alignment, a clash is present between one MeaB helix (residues 206 to 228) and the substrate-binding TIM barrel domain of *Ps*MCM (Fig. 6*A*). This same clash is also present when MeaB:*Me*MCM_cbl_ is superimposed onto an IcmF structure in which the Cbl-binding domain is sitting on top of the substrate-binding TIM barrel domain with the Cbl positioned for catalysis (Fig. 6*B*) (32). In contrast, no clash is present when MeaB:*Me*MCM_cbl_ is superimposed onto an IcmF protomer structure that displays a Cbl-binding domain that is positioned away from the substrate-binding domain, i.e., an open mutase conformation (Fig. 6*C*) (32). Instead of clashing, the MeaB helix (residues 206 to 228) appears wedged between the Cbl-binding and the substrate-binding domains of the open mutase (Fig. 6*C*). Thus, the active conformation of MeaB appears to be structurally compatible with inactive (open) states of mutases and not with active (closed) mutase states. The inactive conformation of MeaB (19), on the other hand, is structurally compatible with the active (closed) state of IcmF (32); superimposition of these structures is possible without any clashes (*SI Appendix*, Fig. S7). There is a chemical logic to these observations; the binding of the active conformation of MeaB should be restricted to inactive mutases that need Cbl.

## Discussion

Metallochaperones assist in the maturation of metalloenzymes, ensuring that valuable metallocofactors are delivered efficiently and with minimal toxicity (35). The molecular basis of metallochaperone function is an active area of research with many open questions (15). In this study, we investigate the molecular mechanism of the AdoCbl maturase MeaB using a minimal system: MeaB in complex with an apo Cbl-binding domain. This minimal system has allowed us to probe the molecular basis of GTP binding and hydrolysis in MeaB, the basis by which MCM_cbl_ binding enhances MeaB’s GTPase activity, and the basis for the conformational opening and closing of the mutase that allows for AdoCbl insertion and holoenzyme maturation.

The structure of GMPPCP-bound MeaB:MeMCM_cbl_ provided the first view of a fully formed active site for MeaB, allowing us to further consider previous proposals regarding the molecular mechanism of GTP hydrolysis. Asp92 of the switch I region has been proposed to activate and position a water molecule for an in-line nucleophilic attack of the γ-phosphate group (19). In the presence of Mg^2+^-GMPPCP, we find that Asp92 is ~4.5 Å (~4.1 Å in the other protomer) away from the oxygen of the γ-phosphate with an approximately in-line orientation (Fig. 4*D*). Thus, Asp92 is a strong candidate to position and activate water for nucleophilic attack. No water is present in the structure near the terminal phosphate of GMPPCP, however. This lack of a water molecule may be due to the fact that GMPPCP is not a perfect mimic of GTP (P–C–P bonds: 112° versus P–O–P bonds: 121°). The structure of MeaB:*Me*MCM_cbl_ also reveals another possible player in catalyzing GTP hydrolysis; Lys188B of the switch III region is ~2.1 Å (~2.4 Å in the other protomer) away from the oxygen of γ-phosphate and ~3.4 Å (~3.0 Å in the other protomer) from Asp92 (Fig. 4*D*). Substitutions of both Asp92 and Lys188 lead to MeaB variants that are impaired in their GAP activity (21, 28).

A key question for many GTPases is how the GTPase is designed such that its activity can be stimulated by the binding of a GAP or target protein. Here, the structure of this minimal system provides insights into the mechanism by which the binding of MeaB to MCM_cbl_ enhances GTP hydrolysis by 25-fold. We find that MeaB’s molecular mechanism involves “inactive” and “active” conformers with MCM binding causing a shift of the conformational equilibrium toward the “active MeaB conformer.” The “active conformer” is stabilized by interactions across the MeaB dimer interface (Asp92A–Lys188B; Arg108A–Asp182B; Glu154A–Lys188B; and Gln160A–Gln185B) and by interactions of switch III residues with the GTP (Asp182B–α-phosphate; Gln185B–γ-phosphate; and Lys188B–γ-phosphate). Almost all of these residues have been subject to mutagenesis previously (D92A, D92N, K188A, K188E, D182A, E154A, Q160A, and Q185A) with substitutions shown to decrease the stimulatory effect of MCM binding on GTP hydrolysis, consistent with a role in stabilizing an active conformer (20, 21, 28). Q185A and K188A, the residues of switch III that directly coordinate the terminal phosphate, have the largest effects on GAP activity (21), and substitutions of the equivalent residues in MMAA, the human homolog of MeaB, have been reported to cause methylmalonic aciduria (29). Notably, our minimal model is not reporting on all of the stabilizing interactions: binding the full mutase stimulates the GTPase activity of MeaB by 100-fold, considerably more than the 25-fold of the Cbl-binding domain alone (9). We believe that this higher GAP activity is due to a gain of interactions between MeaB and the substrate-binding TIM barrel that further help to stabilize the active conformation of MeaB.

**Fig. 7.**
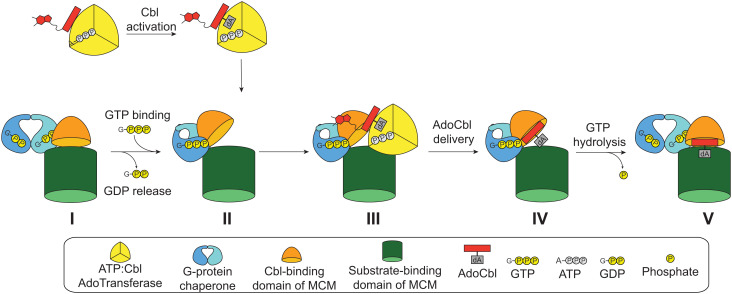
Proposed steps for loading of mutase-active site with AdoCbl. In solution, *Me*MCM associates with GDP bound MeaB. The binding of GTP and the Mg^2+^ ion displace GDP. In the presence of both the mutase and Mg^2+^-GTP bound, MeaB adopts an active conformation that is proposed to wedge open the active site of the mutase. The ATR prepares the AdoCbl and inserts it into the wedged open active site of the mutase. After GTP hydrolysis occurs, the conformation of the MeaB returns to the inactive state and the mutase closes, trapping the AdoCbl. For simplicity, the inactive subunit of the *Me*MCM, which is not predicted to interact with the G-domain (*SI Appendix*, Fig. S9), is omitted.

Additionally, these structural data provide insight into how MeaB facilitates the delivery of AdoCbl to a target mutase protein, either as part of a mutase maturation or a mutase repair process. We observe that when Mg^2+^-GTP binds MeaB (Fig. 7, state I), the second protomer of MeaB rearranges to form an “active conformer.” Structural superimpositions (Fig. 6*C*) suggest that a helix of the second protomer of active MeaB acts as a wedge between the Cbl-binding and substrate-binding domains of the mutase, presumably opening up the mutase for Cbl delivery or stabilizing an open conformation, which is formed by an apo or damaged mutase (Fig. 7, state II). An open mutase conformation would allow for AdoCbl transfer from ATR to MCM (Fig. 7, state II to IV). Once AdoCbl is transferred, the mutase must close down to trap AdoCbl inside and position it for catalysis. Our structural data suggest that GTP hydrolysis will dramatically destabilize the “active conformer,” transitioning MeaB back to the inactive conformation (Fig. 7, state IV to V). Our structure informs us that hydrolysis of GTP would result in the loss of the stabilizing interactions made to the terminal phosphate, to Lys188, and to the Mg^2+^ ion, resulting in the repositioning of Arg108, which in turn should break cross-dimer interactions. In other words, GTP hydrolysis would result in the loss of all the stabilizing interactions that we have identified for the “active conformer.” When MeaB undergoes its 180° rotation back to the “inactive conformer”, the mutase closes and is ready for catalysis (Fig. 7, state V).

Previously, the Banerjee lab showed that substitutions of switch III residues Asp182, Gln185, and Lys188 not only reduced in vitro GAP activity of MCM, but also uncoupled AdoCbl transfer from GTP hydrolysis (21). In these experiments, authors measured AdoCbl transfer from ATR to MCM in presence of MeaB and a nonhydrolyzable analog of GTP, GMPPNP. For wild-type MeaB, their data indicated that in the absence of GTP hydrolysis, AdoCbl is transferred from ATR but is not captured by MCM. Presumably without GTP hydrolysis, the mutase cannot close, so AdoCbl is released into solution. In contrast to wild-type MeaB, variants of MeaB that have a less stable active conformation, including switch III variants D182A, Q185A, and K188A and switch I variants D92A and D105A, can capture more AdoCbl (21, 28). AdoCbl is transferred by ATR and much more of it is captured by MCM. Presumably, in these cases, GTP hydrolysis was not necessary to convert these MeaB variants back to their inactive conformations, releasing the molecular wedge that was holding MCM open. These variants were unstable enough to transition back to the inactive states on their own, allowing for increased AdoCbl capture. Thus, in these MeaB variants, mutase maturation was uncoupled from GTP hydrolysis.

The proposed process of mutase maturation shown in Fig. 7 requires the formation of a MeaB dimer that has one protomer attached to the Cbl-binding domain of the mutase via switch I and the other inserted between Cbl-binding and substrate-binding domains to stabilize an open conformation of the mutase for AdoCbl delivery. This model for MeaB begs the question of whether the already dimeric IcmF needs to form transient higher-order oligomers for AdoCbl insertion, i.e., whether the same active and inactive protomer arrangements observed for MeaB also exist for the chaperone domain of IcmF, and if so, what is the benefit (if any) of the fused IcmF system. This MeaB model also begs the question of how many other dimeric metallochaperones (if any) will employ a similar molecular mechanism. There is considerable structural similarity between NTPase metallochaperones (15), leaving open the possibility that target protein binding will accelerate the GTPase activity of other metallochaperones via 180° rotations. Additional structural data will be invaluable for answering these questions.

Sometimes smaller is better. This minimal system has provided a wealth of information about AdoCbl-dependent mutase maturation. Using this minimal system, we were finally able to provide a molecular explanation for the importance of the switch III region and the basis for methylmalonic aciduria when switch III residues Lys188 and Gln185 are substituted. It is always intriguing to consider the molecular function of metallochaperones that do not directly bind their metallocofactor. What do these chaperones do exactly and what is the role of GTP hydrolysis? Active sites in metalloenzymes are often buried to protect the reactive intermediates and/or to protect the metallocofactor itself from oxidation or other unwanted chemistry. In all mutases, AdoCbl is buried (36), presumably for both reasons. Here, we find that the ability to open up a mutase to insert AdoCbl and then close the structure down again is key to capturing this metabolically expensive cofactor (approximately 30 enzymes are needed for Cbl biogenesis). Metalloenzyme maturation processes can be as fascinating as the metalloenzymes are themselves, and understanding maturation is essential for industrial applications of metalloenzymes and for an understanding of human disease. We hope that the studies presented here will benefit metalloenzyme applications in industry and in medicine by providing insights into metalloprotein maturation.

## Materials and Methods

### Materials.

All chemicals, solvents, and reagents were purchased from Sigma-Aldrich unless otherwise noted below. Restriction enzymes (*NdeI*, *NcoI*, and *XhoI*), *Escherichia coli* Bl21 T7 Express competent cells, and the Gibson Assembly® kit were purchased from New England Biolabs. Sanger sequence verification was performed by Genewiz®. Luria Broth (LB) medium components were purchased from Fisher BioReagents. Kanamycin was purchased from GoldBio and used at a concentration of 50 µg/mL. Isopropyl-β-D-thiogalactopyranoside (IPTG) was purchased from GoldBio. The NaCl for purification buffer components was purchased from Fisher Chemical. The EDTA-free protease inhibitor cocktail tablets were purchased from Roche. The Ni-NTA 1-mL columns and Superdex75 16/60, Superdex200 16/60, and Superdex200 10/300 Increase GL size exclusion columns (SEC) were purchased from GE Healthcare. The gel filtration standards, polyacrylamide gels, sodium dodecyl sulfate solution, Bradford protein assay dye, and bovine serum albumin were purchased from BioRad. The MgCl_2_ was purchased from CalBiochem. The crystallization solution (PEG 3350, LiCl) was purchased as one condition in the 96-condition PEG/Ion High Throughput screen from Hampton Research.

The methods provided here have been described previously in the doctoral dissertation of Dr. David Born (37) and reproduced here with updates and modifications.

### Cloning.

The genes encoding MeaB (WP_003597297.1) and the Cbl-binding domain (residues 545 to 712) of the alpha subunit of MCM (*Me*MCM_cbl_) (WP_015857646.1) from *M. extorquens*, each with an N-terminal hexahistidine affinity tag (HisTag), were synthesized individually by GenScript. Each individual gene was inserted into separate pET28a expression vectors at the *NdeI* and *XhoI* restriction sites.

Preliminary solubility tests of the *Me*MCM_cbl_ with an N-terminal HisTag indicated low solubility, so a pET28a vector containing *Me*MCM_cbl_ with an added C-terminal HisTag was constructed to improve *Me*MCM_cbl_ solubility. The *Me*MCM_cbl_ gene was amplified from the pET28a vector with appropriate extension primers to facilitate Gibson assembly (38) into a pET28a vector cut with restriction enzymes *NcoI* and *XhoI*. The sequences were verified by Sanger sequencing.

### Primers for Gibson Assembly

**Table:** 

Name	Sequence (5′ to 3′)
pET28a_*Me*MCM_Cbl_Fw_1 (PCR amplification)	ATGCGTGCGCAGATCCGTAG
pET28a_*Me*MCM_Cbl_Rv_1 (PCR amplification)	CAGACGGGTGTTCAGTTCACCC
pET28a_*Me*MCM_Cbl_Fw_2 (installation of C-terminal HisTag)	GAAATAATTTTGTTTAACTTTAAGAAGGAGATATACCATGCGTGCGCAGATCCGTAG
pET28a_*Me*MCM_Cbl_Rv_2 (installation of C-terminal HisTag)	GATCTCAGTGGTGGTGGTGGTGGTGCTCGAGCAGACGGGTGTTCAGTTCAC

Plasmids

**Table:** 

Name	Features	Source
pET28a-MeaB	KAN^R^_,_ *NdeI,* and *XhoI* restriction sites	This study
pET28a-*me*MCM_cbl_-N-TerminalHisTag	KAN^R^_,_ *NdeI,* and *XhoI* restriction sites	This study
pET28a-*me*MCM_cbl_-C-terminalHisTag	KAN^R^_,_ *NcoI,* and *XhoI* restriction sites	This study

### Protein Expression and Purification.

Cell growth and purifications of MeaB and *Me*MCM_cbl_ were conducted following the same procedure described here. An overnight starter culture of 100 mL LB supplemented with 50 µg/L kanamycin was inoculated from a single colony of *E. coli* Bl21 T7 Express competent cells transformed with the appropriate gene and grown at 37 °C under shaking. The overnight starter culture was used to inoculate 1 L of LB supplemented with 50 µg/L kanamycin at 37 °C under shaking. The 1 L culture was induced with 0.5 mM IPTG when OD_600_ reached ~0.4 to 0.6 and grown for 16 h at 18 °C under shaking. The cells were harvested by centrifugation (4,000 × g, 15 min, 4 °C) and flash-frozen in liquid N_2_ before being stored in a −80 °C freezer for future use.

Cells from 1 L culture were resuspended in 80 mL lysis buffer (50 mM HEPES pH 7.5, 500 mM NaCl, 25 mM imidazole) supplemented with 1 mM phenylmethylsulfonyl fluoride and 1 Roche EDTA-free protease inhibitor cocktail. Cells were lysed by ultrasonication, and cell lysates were clarified by centrifugation (25,000 × g, 30 min, 4 °C). Clarified lysate was passed through a 0.2-µm filter before being loaded onto a 1-mL Ni-NTA column equilibrated with lysis buffer. Protein was eluted with a linear gradient of elution buffer (50 mM HEPES pH 7.5, 500 mM NaCl, 500 mM imidazole) using an FPLC system (Amersham Biosciences AKTA FPLC System). Elution fractions were concentrated in either a 30-kDa molecular weight cutoff (MWCO) centrifugal filter (for MeaB) or a 10-kDa MWCO centrifugal filter (for *Me*MCM_cbl_). The concentrated fractions of MeaB were loaded onto a Superdex 200 16/60 SEC equilibrated with SEC buffer (50 mM HEPES pH 7.5, 500 mM NaCl). The concentrated fractions of *Me*MCM_cbl_ were loaded onto a Superdex75 16/60 SEC equilibrated with SEC buffer. Elution fractions from SEC were concentrated in a 30-kDa or 10-kDa MWCO centrifugal filter for MeaB or *Me*MCM_cbl_, respectively. Purity was assessed by 4 to 20% (w/v) SDS-PAGE. The concentration of MeaB monomer was determined to be 994 µM (36.4 mg/mL) by UV/Vis absorbance at 280 nm using an extinction coefficient of 23,490 M^−1 ^cm^−1^, determined using the ProtParam tool (39). The concentration of *Me*MCM_cbl_ monomer was determined to be 307 µM by Bradford assay, using bovine serum albumin as a standard (40). Protein samples in SEC buffer were flash-frozen in liquid N_2_ and stored in a −80 °C freezer for future use.

### GTPase Assays.

The GTPase activity of MeaB (2 µM) was determined in the presence of 500 µM GTP. The effect of *Me*MCM_cbl_ (4 µM) in SEC was determined by preincubating the complex (2 µM MeaB) before initiating the GTPase assay with 500 µM GTP. The EnzCheck phosphate assay kit was used for all GTPase assays following the manufacturer’s instructions with the following modifications (Molecular Probes). The assay reactions (200 µL) were prepared excluding the GTP and incubated at room temperature for 30 min before initiating the assay to control for contaminating phosphate in the various components. Assays were performed in triplicate. The absorbance at 360 nm for each assay reaction was recorded using a SpectraMax Plus 384 microplate reader (Molecular Dimensions). The absorbances were converted into concentration of inorganic phosphate using the standard curve generated according to the manufacturer’s directions. The no-enzyme condition was subtracted from the reaction mixtures to control for background GTP hydrolysis.

### Analytical Size-Exclusion Chromatography.

A Superdex200 Increase 10/300 GL SEC column equilibrated with SEC buffer was used to assess complex formation between MeaB and *Me*MCM_cbl_. A 300 µL volume of the following five samples were injected onto the SEC column. Sample 1 contained 50 µM MeaB in SEC buffer. Sample 2 contained 50 µM MeaB and 100 µM *Me*MCM_cbl_ in SEC buffer. Samples 3 to 5 contained 50 µM MeaB and 100 µM *Me*MCM_cbl_ supplemented with 500 µM of the appropriate nucleotide [GDP, guanosine-5′-[(β,γ)-imido]triphosphate trisodium salt hydrate (GMPPNP), or guanosine-5′-[(β,γ)-methyleno]triphosphate sodium salt (GMPPCP)] and 1 mM MgCl_2._ The identity of each elution peak was determined by SDS-PAGE analysis (*SI Appendix*, Fig. S1). The estimated molecular weights of elution peaks were calculated by comparison to the gel filtration standards.

### Crystallography.

The complex between MeaB and *Me*MCM_cbl_ was prepared by incubating equimolar amounts of MeaB and *Me*MCM_cbl_ (207 µM of each protein monomer) in SEC buffer with 1 mM GMPPCP and 2 mM MgCl_2_ for 1 h on ice. The MeaB:*Me*MCM_cbl_ complex was purified by SEC on a Superdex200 16/60 column equilibrated with a SEC buffer. The complex eluted with a molecular weight of approximately 110 kDa, representing a complex of two MeaB protomers and two *Me*MCM_cbl_ protomers. Following SEC, the complex was concentrated using a 30-kDa MWCO centrifugal filter to 182 µM as determined by UV/Vis absorbance at 280 nm using an extinction coefficient of 27,960 M^−1 ^cm^−1^_,_ which was calculated by adding the extinction coefficients of MeaB (23,490 M^−1^ cm^−1^) and *Me*MCM_cbl_ (4,470 M^−1^ cm^−1^). The latter value was estimated using the ProtParam tool (39).

A 96-well plate of sitting-drops was set up with a Phoenix liquid handling robot (Art Robbins Instruments) with a Hampton Research PEG/Ion HT screen. The sitting drops were stored and imaged using the Formulatrix® Rock Imager 1000 at 18 °C. Crystals were obtained by the vapor diffusion method by mixing 150 nL of protein solution [182 μM (20.2 mg/mL) complex in SEC buffer supplemented with 182 μM GMPPCP and 364 µM MgCl_2_] with 230 nL precipitant solution [20% (w/v) PEG 3350 and 200 mM LiCl] incubated over 70 μL precipitant solution. A single thick plate-like crystal formed within 9 d and grew to a maximum size of approximately 250 µm within 2 wk. The crystal was broken, and the resulting smaller crystals were transferred stepwise through three drops of increasing glycerol concentration into a cryogenic solution containing 20% (w/v) PEG 3350, 200 mM LiCl, 200 µM GMPPCP, 400 µM MgCl_2_, and 20% (v/v) glycerol and flash-frozen in liquid N_2_.

### Data Collection, Processing, Structure Determination, and Refinement.

A preliminary dataset for MeaB:*Me*MCM_cbl_ was collected on an in-house Cu-K_α_ rotating anode source (Rigaku) with a Saturn 944 CCD detector at a temperature of 100 K in a single 180° wedge with 0.5° per image. Data were indexed, integrated, and scaled in XDS (41).

The structure was solved by molecular replacement with the Phenix implementation of Phaser (42) using data trimmed to 3.1 Å resolution. Two MeaB (PDB 2QM7) (19) protomers with the C-terminal dimerization helices removed (residues 5 to 293 out of 329) were first placed individually to create a partial solution which was used as a starting point for molecular replacement to place the Cbl-binding domains. The Phenix implementation of Sculptors (43), which removed the sidechains of divergent sequences, was used to create a homology model of *Me*MCM_cbl_ using the Cbl-binding domain of MCM (residues 545 to 712) from *Propionibacterium freudenreichii subsp. shermanii* (PDB 4REQ) (25) (70.9% identical). The final molecular replacement solution with LLG of 210 and TFZ of 12.0 identified four protomers in the asymmetric unit, corresponding to two MeaB protomers (chains A and B) and two *Me*MCM_cbl_ protomers (chains C and D).

Applying noncrystallographic symmetry (NCS) restraints to each of the chains of the same protomer, the final solution identified from molecular replacement was refined using phenix.refine (44) with two rounds of simulated annealing at 5,000 K to minimize model bias. Additionally, rigid body, positions, and group *B*-factors were refined with coordinate restraints (wc) equal to four and *B-*factor restraints (wu) equal to three and the weighting of the crystallographic refinement target (wxc_scale) equal to 0.1 in order to optimize the weight of geometry restraints compared to X-ray data. Subsequent iterative rounds of model building and refinement were performed in Coot (45) and Phenix, respectively. Sidechains were added to residues with clear electron density, and one molecule of GMPPCP was modeled in for each MeaB protomer. Iterative refinement including positional and group *B*-factor refinement with NCS restraints continued until the *R*-factors were 32.5% and 33.4% for the working *R*-factor (*R*_work_) and the free *R-*factor (*R*_free_) representing 5% of reflections, respectively. This model was not refined to completion and only used as a starting model for the additional dataset (*SI Appendix*, Table S1).

An additional dataset for the same MeaB:*Me*MCM_cbl_ crystal used for the preliminary model was collected at the Advanced Photon Source (Argonne, Illinois, USA) on beamline 24ID-E using an Eiger-16M pixel array detector at a temperature of 100 K. Data were collected on the MeaB:*Me*MCM_cbl_ crystal at a wavelength of 0.9791 Å in a single 360° wedge with 0.25° per image with 5% transmission. The MeaB:*Me*MCM_cbl_ crystal belongs to space group *P*2_1_2_1_2_1._ The MeaB:*Me*MCM_cbl_ data were indexed, integrated, and scaled to 2.72 Å resolution in XDS. The same *R*-free flags from the preliminary dataset were used and extended in CCP4 (46).

With NCS restraints for each of the chains of the same protomers, the model was refined with one round of simulated annealing at 2,000 K to minimize model bias. Additionally, rigid body, positional and group *B*-factor refinement were used. Side chains were added to residues with clear electron density and one Mg^2+^ ion was placed with each MeaB protomer, restraining the distances from Mg^2+^ to its ligands to 2.1 Å. Waters were placed manually into regions with 2mF_o_-DF_c_ composite omit density, 2F_o_-F_c_ density, and 3 σ F_o_-F_c_ density. Restraints from the CCP4 monomer library were used for the GMPPCP. The placement of GMPPCP, glycerol, and waters were confirmed using a 2mF_o_-DF_c_ composite omit map. The crystallographic refinement target for coordinates (wxc_scale), the crystallographic refinement target for *B*-factors (wxu_scale), coordinate restraints (wc), and *B-*factor restraints (wu) were optimized on each round of refinement. Subsequent iterative rounds of model building and refinement were performed in Coot and Phenix, respectively. The final model has a *R*_work_ of 21.0% and *R*_free_ of 23.6%. In chain A, residues Thr4-Gly294 and Val298-Ile327 of 329 were modeled into the density. In chain B, residues Met1-Pro228, Trp233-Leu262, and Asp276-Leu329 of 329 were modeled into the density. In chain C, residues Ser563-Gly592 and Gly595-Leu712 of 712 were modeled into the density along with two residues of the C-terminal HisTag (713 to 714). In chain D, residues Ser563-Thr710 of 712 were modeled into the density. There was no density observed for the affinity tags on chains A, B, or D. Crystallographic software packages were compiled by SBGrid (47) (*SI Appendix*, Table S1). Structural figures were made in PyMOL 2.3.3 (The PyMOL Molecular Graphics System Version 2.3.3 Schrodinger, LLC).

## Supplementary Material

Appendix 01 (PDF)

## Data Availability

Crystallographic coordinates data have been deposited in [protein data bank] (PDB ID 8DPB) (48).
