# Supplementary material for: Structure of metallochaperone in complex with the cobalamin-binding domain of its target mutase provides insight into cofactor delivery
Source: Proc Natl Acad Sci U S A. 2023 Feb 14;120(8):e2214085120. doi: 10.1073/pnas.2214085120 (PMC9974510; doi:10.1073/pnas.2214085120)
Supplement: Supplementary file 1 — Appendix 01 (PDF) [file pnas.2214085120.sapp.pdf]

Supporting Information for
Structure of metallochaperone in complex with the cobalamin-binding domain of its target mutase provides insight into cofactor delivery

Francesca A. Vaccaro, David A. Born, Catherine L. Drennan*

*Corresponding author: C.L. Drennan
Email: cdrennan@mit.edu

This PDF file includes:

Figures S1 to S9
Tables S1 to S4
SI References

Supporting Figures

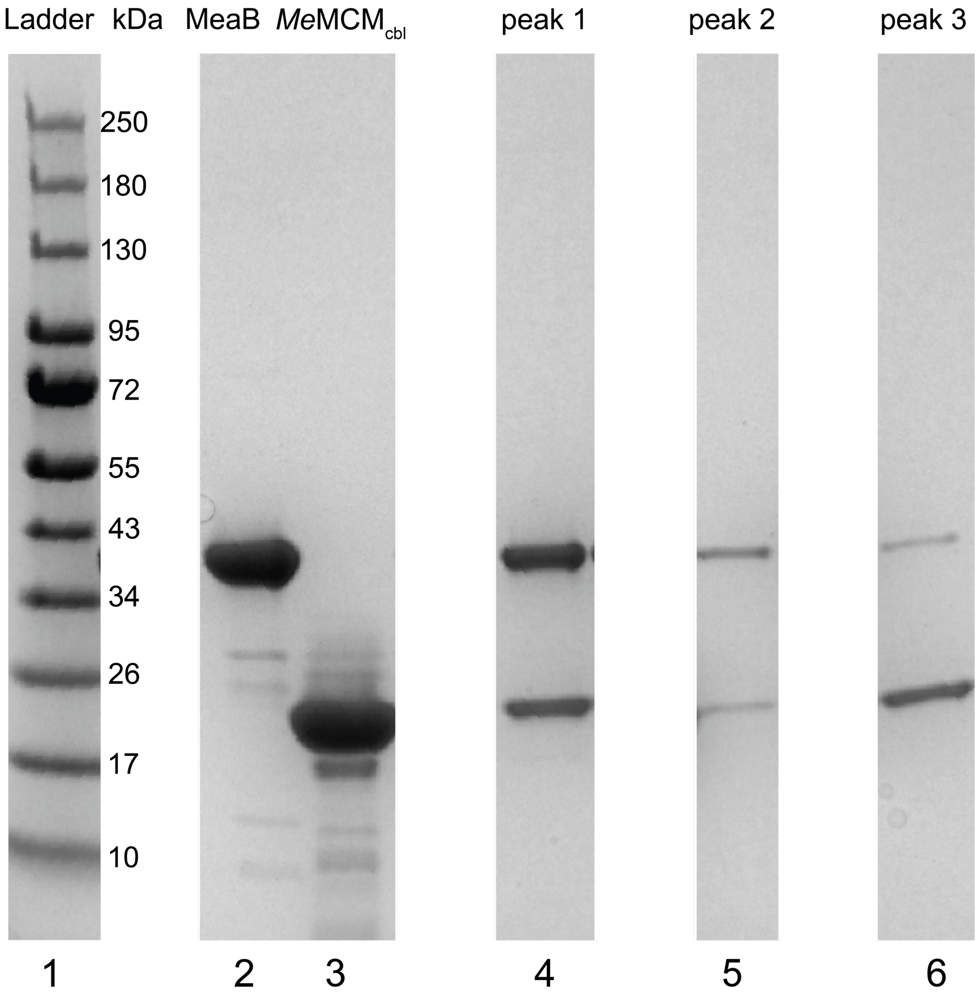

Figure S1. SDS-PAGE Analysis Confirms the Presence of the Complex of MeaB with *Me*MCM$_{cbl}$. Each peak from the analytical SEC of MeaB, *Me*MCM$_{cbl}$ and GMPPCP was loaded onto an SDS-PAGE gel and compared to samples of MeaB and *Me*MCM$_{cbl}$. Lane 1, Ladder. Lanes 2 and 3, MeaB and *Me*MCM$_{cbl}$, respectively. Lane 4, elution peak 1 at 13.5 minutes which contains both MeaB and *Me*MCM$_{cbl}$ in equal amounts. Lane 5, elution peak 2 at 14.5 minutes which contains primarily MeaB. Lane 6, elution peak 3 at 16.5 minutes which contains primarily *Me*MCM$_{cbl}$.

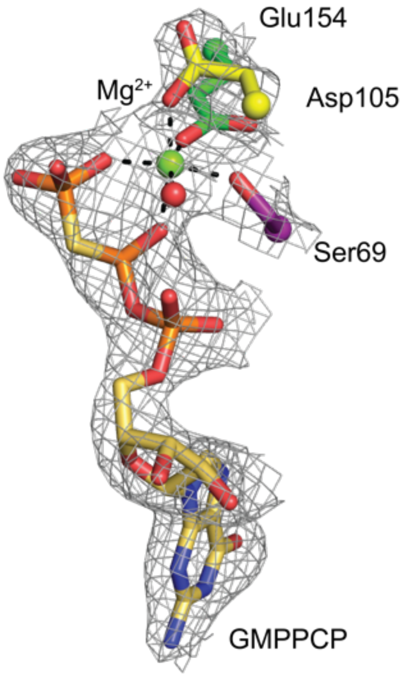

Figure S2. Composite Omit Density Confirms the Presence of a Mg^{2+} ion in MeaB in the MeaB:*Me*MCM$_{cbl}$ structure. The $2mF_o$-DF_c composite omit maps contoured to 1σ confirm that Mg^{2+} ion (green sphere) is coordinated by a water (red sphere), the oxygen atoms of Ser69, Asp105, and Glu154 from MeaB, and the β- and γ-phosphate groups of GMPPCP.

```
CmIcmF      ------------------------------------------------------------      168
MeMeaB      ------------------------------------------------------------      0
HsMMAA      MPMLLPHPHQHFLKGLLRAPFRCYHFIFHSSTHLGSGIPCAQPFNSLGLHCTKWMLLSDG      60


CmIcmF      -----------------------------LDTVVAGDRRAA-QLITALENGKADPE----      195
MeMeaB      -----------------MSATLPDMDTLRERLLAGERAALARAITLAESRRADHRAAVR      42
HsMMAA      LKRKLCVQTTLKDHTEGLSDKEQRFVDKLYTGLIQGQRACLAEAITLVESTHSRKKELAQ      120
                                            :: *:* .  . **  *. ::  .

                                           P-loop
CmIcmF      -LVSAL---H-----AQAKAAAVPVLGITGTGGAGKSSLTDELIRRFRLDQDDALSIAVI      246
MeMeaB      DLIDAVL----------PQTGRAIRVGITGVPGVGKSTTIDALGSLL---TAAGHKVAVL      89
HsMMAA      VLLQKVLLYHREQEQSNKGKPLAFRVGLSGPPGAGKSTFIEYFGKML---TERGHKLSVL      177
             *:. :                .  :*::*  *.***:  : :   :      . .::*:

                   Switch I
CmIcmF      SIDPSRRKSGGALLGDRIRMNAIN-HPNIFMRSLATREAGSEISQALPDVIAACKAARFD      305
MeMeaB      AVDPSSTRTGGSILGDKTRMARLAIDRNAFIRPSPSSGTLGGVAAKTRETMLLCEAAGFD      149
HsMMAA      AVDPSSCTSGGSLLGDKTRMTELSRDMNAYIRPSPTRGTLGGVTRTTNEAILLCEGAGYD      237
            ::***   :**::***: **  :  . * ::*   :  : . ::    :.:  *:.* :*

              Switch II                Switch III         Base Specificity Loop
CmIcmF      LVIVETSGIGQGDAAIVPHVDLSLYVMTPEFGAASQLEKIDMLDFADFVAINKFDRKGA-      364
MeMeaB      VILVETVGVGQSETAVADLTDFFLVLMLPGAGDELQGIKKGILELADMIAVNKADDGDGE      209
HsMMAA      IILIETVGVGQSEFAVADMVDMFVLLLPPAGGDELQGIKRGIIEMADLVAVTKSD-GDLI      296
            ::::** *:**.: *:.  .*: : :: *  *   *  * .::::**::*:.* *  .

CmIcmF      ------QDAWRDVAKQVQRNREQWHSRAEDMPVYGTQASRFNDDGVTMLYQGLVGALGAR      418
MeMeaB      RRANAAASEYRAALHILTPPSATWTPPVVTIS-------GLHGKGLDSLWSRIEDH---R      259
HsMMAA      VPARRIQAEYVSALKLLRKRSQVWKPKVIRIS-------ARSGEGISEMWDKMKDF---Q      346
                     :  . : :      *   .  :           ..*:  ::. : .    :

CmIcmF      GMSLKPGTL----------------------------------PNLEGRISTGQNV----      440
MeMeaB      AKLTATGEIAGKRREQDVKWMWALVHERLHQRLVGSAEVRQATAEAERAVAGGEHS----      315
HsMMAA      DLMLASGELTAKRRKQQKVWMWNLIQESVLEHFRTHPTVREQIPLLEQKVLIGALSPGLA      406
                  * :                                     *  :  *

CmIcmF      -IV---------      442
MeMeaB      ------------      315
HsMMAA      ADFLLKAFKSRD      418
```

Figure S3. Sequence Alignment of MeaB with MMAA and the G-protein Domain of *Cm*IcmF. The regions which participate in binding and catalysis of the nucleotide substrate are highlighted in boxes corresponding to their coloring in the structure of MeaB in Figure 1. Asterisks (*) denote positions with conserved residues. Colons (:) denote positions with conservation with strongly similar properties. Periods (.) denote positions with conservation with weakly similar properties. Alignments performed using ClustalW (1).

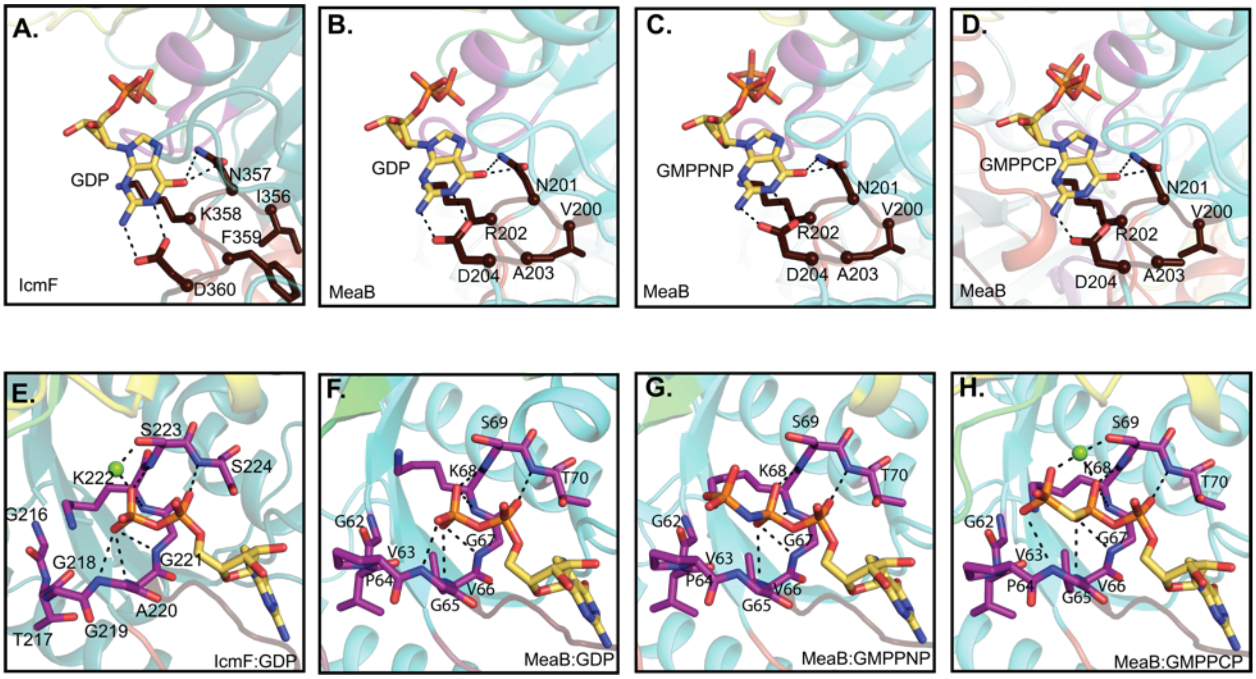

Figure S4. The Base Specificity Loop and the P-loop Make Similar Interactions in all MeaB structures and in the IcmF structure. (A) GDP-bound IcmF structure (PDB 4XC6) with base specificity loop (brown sticks) of IcmF (cyan ribbons) providing stabilizing hydrogen bonds to the nucleotide. Asp360 coordinates both the amine at position 2 and the nitrogen at position 1, and Asn357 coordinates the carbonyl at position 6 to specifically bind only guanine bases. (B) GDP-bound MeaB structure (PDB 2QM7) with base specificity loop of MeaB providing stabilizing hydrogen bonds to the nucleotide. Asp 204 corresponds to Asp360 in IcmF; Asn201 corresponds to Asn357 in IcmF. Colored as in A. (C) GMPPNP-bound MeaB structure (PDB 4JYB). Colored as in A. (D) GMPPCP-bound MeaB:*Me*MCM$_{cbl}$ structure (this work). Colored as in A. (E) GDP-bound IcmF structure (PDB 4XC6) with the amines of the backbone of the P-loop (purple sticks) providing stabilizing hydrogen bonds to the negatively charged phosphate groups. (F) GDP-bound MeaB structure (PDB 2QM7) with the corresponding P-loop residues in MeaB shown as sticks. Colored as in E. (G) GMPPNP-bound MeaB structure (PDB 4YJB). Colored as in E. (H) GMPPCP-bound MeaB:*Me*MCM$_{cbl}$ structure (this work). Colored as in E. The nucleotides are shown as yellow sticks in all panels.

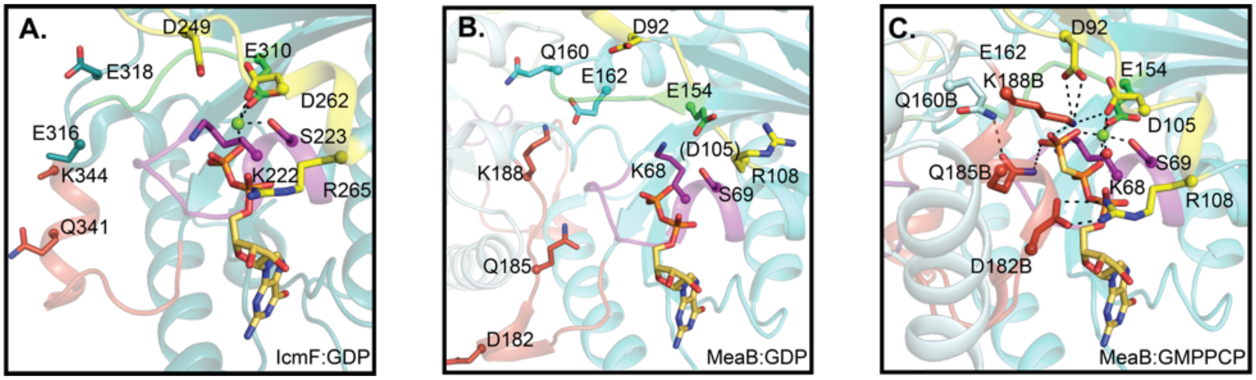

Figure S5. The Nucleotide Binding Site of IcmF Compared to MeaB. (A) The GDP-bound G-protein domain of IcmF (PDB 4XC6). Coloring: P-loop (purple), switch I (yellow), switch II (green), switch III (red orange), additional residues that are outside of the motifs that undergo conformational rearrangements (teal for IcmF, cyan for MeaB). Base specificity loop interactions are shown in **Figure S4** instead of here for clarity. Mg^{2+} ion in green. (B) MeaB with GDP as shown in Fig. 4. (C) GMPPCP-bound MeaB in the presence of *Me*MCM$_{cbl}$ (this work, also shown in Fig. 4). The "B" label indicates residues from MeaB chain B.

```
CmIcmF          ----------MTDLSDVSRTAAAKPPAVPGRGPANKVRFVTAASLFDGHDASINIMRRIL   50
HsMCM           SGAYRQEFGE-SKEITSAIKRVHKFMEREGR----RPRLLVAKMGQDGHDRGAKVIATGF   638
MeMCM           SGVYKREVGGMSPVVEKVRGLVEAFEENDGR----RPRILVAKMGQDGHDRGQKVIASAF   607
PsMCM           SGVYSKEVKN-TPEVEEARELVEEFEQAEGR----RPRILLAKMGQDGHDRGQKVIATAY   621
                           :         .       **    : *:: *    **** . :::

CmIcmF          QSQGCEVIHLGHNRSVQEVVTAALQEDVQGIAISSYQGGHVEYFKYMIDLLREHGGEHIQ   110
HsMCM           ADLGFDVDIGPLFQTPREVAQQAVDADVHAVGISTLAAGHKTLVPELIKELNSLGRPDIL   698
MeMCM           ADLGFDVDIGPLFATPDEAARQAVENDVHIVGVSSLAAGHLTLVPELKAALKQEGRDDVM   667
PsMCM           ADLGFDVDVGPLFQTPEETARQAVEADVHVVGVSSLAGGHLTLVPALRKELDKLGRPDIL   681
                 . * :*       :  *..  *:: **: :..:*: .**   .  :   * . *  .:

CmIcmF          VFGGGGGVIVPDEIRELQAYGVARIYSPEDGQRMGLAGMITDMAQRCDIDLTRYAPTT     168
HsMCM           VM--CGGVIPPQDYEFLFEVGVSNVFGPGTRIPKAAVQVLDDIEKCLEKKQQSV----     740
MeMCM           IV--VGGVIPPGDYDALYAAGASAIFPPGTVIAEAAVKLLGELNTRLGYGERQAAE--     721
PsMCM           IT--VGGVIPEQDFDELRKDGAVEIYTPGTVIPESAISLVKKLRASLDA---------     728
                :    ****   :   *   *.   :: *      .   :: .:
```

Figure S6. Sequence alignment of the Cbl-binding domain of *Cm*IcmF, *Hs*MCM, *Me*MCM, and *Ps*MCM. The sequences aligned are residues 1-168 from *Cm*IcmF, residues 584-740 from *Hs*MCM, residues 552-721 from *Me*MCM active subunit, and residues 567-728 from *Ps*MCM active subunit. Asterisks (*) denote positions with conserved residues. Colons (:) denote positions with conservation with strongly similar properties. Periods (.) denote positions with conservation with weakly similar properties. Alignments performed using ClustalW(1).

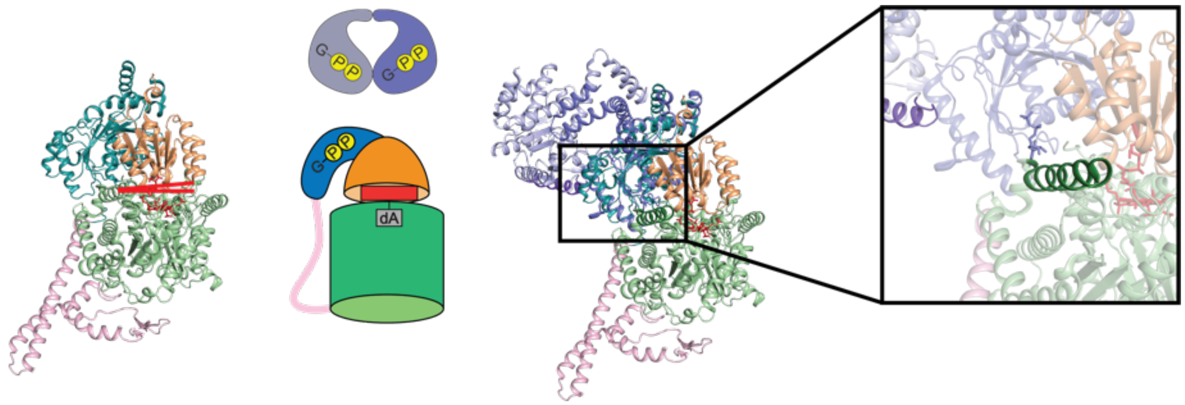

Figure S7. Structural Superimposition of the Previous MeaB Structure with Full-length IcmF. (left) IcmF (PDB 4XC6) with substrate-binding domain (light green), Cbl-binding domain (light orange), G-protein domain (teal), and linker (pink). Red lines indicate that there is no gap between domains for Cbl insertion, i.e. that IcmF is in the active, closed state. (middle) Cartoon of "inactive conformer" of MeaB bound to GDP and a closed protomer of IcmF. (right) Overlay of the GDP-bound "inactive conformer" of MeaB (purple and light purple) with the closed IcmF protomer structure. Insert shows that the "inactive conformer" of MeaB can bind to the active IcmF protomer with no clashes.

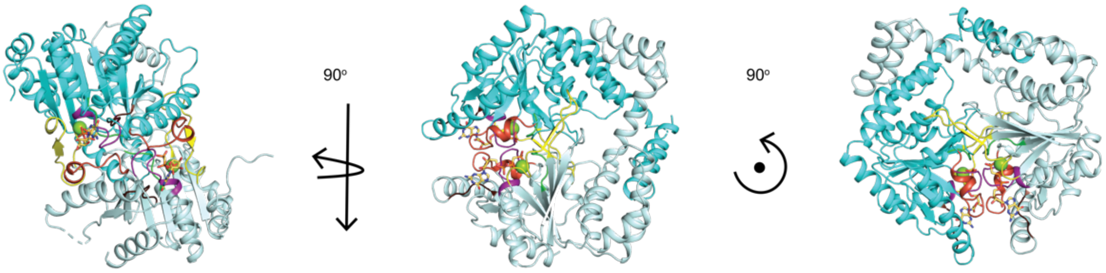

Figure S8. Comparison of the Views of MeaB. The active conformation of MeaB (chain A: light blue, chain B: cyan) has a new interface comprised of the switch III region (red orange). The base specificity loop (dark brown), P-loop (purple), switch I (yellow), switch II (green) regions interact with the Mg^{2+} ions (green spheres) and GMPPCP (yellow sticks) but are buried from the surface and inaccessible to the mutase. Rotating the active conformation (left) around the y-axis 90˚ (middle) and then another 90˚ around the z-axis (right) yields the side view of MeaB shown in Figure 1A, 3A and 3B.

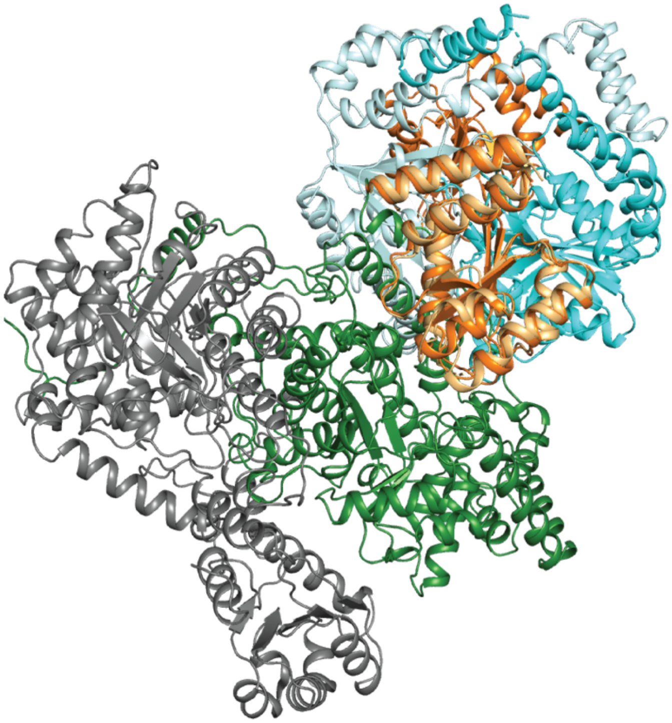

Figure S9. Structural Superimposition of *Ps*MCM with MeaB:*Me*MCM$_{cbl}$:GMPPCP Suggests that MeaB Does Not Interact with the Inactive Subunit of MCM. When Cbl-binding domains of *Ps*MCM (PDB 1REQ) and MeaB:*Me*MCM$_{cbl}$:GMPPCP (orange) are superimposed, MeaB (cyan) contacts the active substrate-binding domain of *Ps*MCM (dark green) but not the inactive subunit of *Ps*MCM (gray).

Supporting Tables

Table S1. Data collection and refinement statistics for MeaB:*Me*MCM$_{cbl}$

**Data Collection**		
Beamline	Cu$_{\alpha}$ Home Source*	APS 24-ID-E
Wavelength (Å)	1.54	0.9792
Space group	$P2_12_12_1$	$P2_12_12_1$
Cell dimensions, *a, b, c* (Å)	66.1, 81.0, 166.2	66.2, 81.0, 166.4
Resolution (Å)	45.72-3.10	45.76-2.72
R_{meas} † (%)	27. 4 (103.7)	15.8 (68.9)
<*I*>/<σI>	7.5 (2.2)	10.9 (2.5)
Completeness (%)	94.2 (100.0)**	99.6 (99.0)
Redundancy	7.1 (8.0)**	8.4 (8.4)
CC1/2	98.7 (80.1)	99.6 (80.4)
No. Total Reflections	112637 (8942)	206565 (15753)
No. Unique Reflections	15835 (1120)	24685 (3897)
**Refinement**		
Resolution (Å)	16.28-3.10	48.99-2.72
No. Reflections used	15696 (1626)	24625 (2366)
R_{work}/R_{free}‡	29.8/31.1	21.0/23.6
No. molecules in asymmetric unit	4	4
MeaB protomers	2	2
*Me*MCM$_{cbl}$ protomers	2	2
No. atoms		
Protein	6464	6501
GMPPCP	64	64
Glycerol	-	12
Mg^{2+}	-	2
Water	-	67
B-factors (Å^2)		
Protein		
Chain A	39.4	48.8
Chain B	40.5	49.7
Chain C	50.2	59.4
Chain D	39.8	51.3
GMPPCP	36.5	36.3
Glycerol	-	53.8
Mg^{2+}	-	33.8
Water	-	39.2
RMS deviations		
Bond lengths (Å)	0.027	0.012
Bond angles (°)	2.64	0.90
Rotamer outliers (%)	26.5	1.66
Ramachandran plot (%)		
Most favored	90.0	98.8
Additionally allowed	6.6	1.2
Disallowed	3.4	0.0

*This model was not refined to completion. **Data are more complete and more redundant in high resolution bin than overall due to loss of data because of ice rings

† Values in parentheses are for the highest resolution shell.

‡5% of reflections were set aside for a test set

Table S2. Interactions of the switch III residues in the MeaB:*Me*MCM$_{cbl}$:GMPPCP complex

	MeaB Chain B	Dist. [Å]	MeaB Chain A		MeaB Chain A	Dist. [Å]	MeaB Chain B
1	Asp182[OD1]	2.5	Arg108 [NH2]	1	Asp182[OD1]	2.6	Arg108 [NH2]
2	Asp182[OD2]	3.5	Arg108 [NH1]	2	Asp182[OD2]	3.5	Arg108 [NH1]
3	Gln185[NE2]	3.5	GMPPCP[O1G]	3	Gln185[NE2]	3.1	GMPPCP[O1G]
4	Lys188[NZ]	2.1	GMPPCP[O1G]	4	Lys188[NZ]	2.4	GMPPCP[O1G]
5	Lys188[NZ]	3.4	Asp92[OD1]	5	Lys188[NZ]	3.0	Asp92[OD1]

Table S3. Hydrogen bonds between MeaB and *Me*MCM$_{cbl}$ in the MeaB:*Me*MCM$_{cbl}$:GMPPCP complex

	MeaB Chain B	Dist. [Å]*	*Me*MCM$_{cbl}$
1	Arg20 [NH2]	2.9	D:Glu632 [OE2]
2	Ala21 [O]	3.7	D:Arg663 [NH2]
3	Arg25 [NH2]	3.3	D:Asp634 [OD1]
4	Arg25 [NE]	3.0	D:Asp634 [OD2]
5	Thr28 [OG1]	2.8	D:Asp634 [OD2]
6	Thr28 [OG1]	3.7	D:Arg582 [NH1]
7	Ser32 [OG]	3.8	D:Gly581 [O]
8	Arg33 [N]	3.0	D:Gly581 [O]
9	Thr98 [O]	3.7	D:Arg583 [NH1]
10	Thr98 [OG1]	3.1	D:Ala608 [O]
11	Ser101 [N]	3.1	D:Phe612 [O]
12	Ser101 [N]	3.9	D:Asp613 [OD1]
13	Ser101 [OG]	2.7	D:Asp613 [OD1]
14	Ser101 [O]	3.0	D:Val614 [N]
15	Ler103 [N]	2.8	D:Val614 [O]
16	Leu103 [O]	3.4	D:Ile616 [N]
17	Lys106 [NZ]	2.8	D:Glu632 [OE1]
18	Lys106 [NZ]	3.0	D:Asn633 [OD1]
19	Thr107 [O]	3.8	D:His596 [NE2]
20	Thr107 [OG1]	2.5	D:Ile616 [O]
21	Ala113 [O]	2.7	D:Arg628 [NH1]
22	Asp115 [O]	3.3	D:Arg628 [NH1]
23	Asp115 [O]	3.5	D:Arg628 [NH2]
24	Arg121 [NH1]	3.3	D:Glu632 [O]
25	Pro122 [O]	3.0	D:Arg585 [NH2]
26	Lys189 [NZ]	2.8	C:Asp609 [OD1]
27	Lys189 [NZ]	3.6	C:Ser605 [OG]

* Interactions calculated using PDBe PISA v1.52 (2)

Table S4. Salt bridges between MeaB and *Me*MCM$_{cbl}$ in the MeaB:*Me*MCM$_{cbl}$:GMPPCP complex

	MeaB Chain B	Dist. [Å]*	*Me*MCM$_{cbl}$
1	Arg20 [NE]	3.8	D:Glu632 [OE2]
2	Arg20 [NH2]	2.9	D:Glu632 [OE2]
3	Arg25 [NH2]	3.3	D:Asp634 [OD1]
4	Arg25 [NE]	3.3	D:Asp634 [OD1]
5	Arg25 [NE]	3.0	D:Asp634 [OD2]
6	Arg33 [NE]	3.2	D:Glu577 [OE2]
7	Lys106 [NZ]	2.8	D:Glu632 [OE1]
8	Lys189 [NZ]	2.8	C:Asp609 [OD1]

* Interactions calculated using PDBe PISA v1.52 (2)

SI References

1. F. Sievers *et al.*, Fast, scalable generation of high-quality protein multiple sequence alignments using Clustal Omega. *Molecular Systems Biology* **7**, 539 (2011).
2. E. Krissinel, K. Henrick, Inference of macromolecular assemblies from crystalline state. *Journal of molecular biology* **372**, 774-797 (2007).
